# Parasitoid-induced changes in metabolic rate and feeding activity of the emerald ash borer (Coleoptera: Buprestidae): implications for biological control

**DOI:** 10.1038/s41598-023-50147-8

**Published:** 2023-12-19

**Authors:** Ying-Qiao Dang, Jian J. Duan, Andrew Y. Li

**Affiliations:** 1 Agriculture Research Service, Beneficial Insects Introduction Research Unit, U.S. Department of Agriculture, Newark, DE 19713 USA; 2 Ecology and Nature Conservation Institute, Chinese Academy of Forestry, Beijing, 100091 China; 3 Agriculture Research Service, Invasive Insect Biocontrol and Behavior Laboratory, U.S. Department of Agriculture, Beltsville, MD 20705 USA

**Keywords:** Ecology, Physiology

## Abstract

Parasitoid-host interactions form the foundation of biological control strategies against many agriculture and forest insect pests. The emerald ash borer (EAB), *Agrilus planipennis* (Coleoptera: Buprestidae), is a serious invasive pest of ash (*Fraxinus* spp.) trees in North America. *Tetrastichus planipennisi* (Hymenoptera: Eulophidae) is a gregarious, koinobiont endoparasitoid, attacking late (3rd to 4th) instars of EAB larvae, which feed in the live phloem of ash trunks or branches, making serpentine-like galleries filled with larval frass. In the present study, we tested the hypothesis that *T. planipennisi* regulates the host metabolism and feeding activity to optimize its offspring development and fitness. We first compared the respiration rate of parasitized and unparasitized host larvae at different times after parasitism, and then measured feeding activity of both parasitized and unparasitized host larvae inside their feeding galleries. Although parasitized host larvae increased metabolic rate and feeding activity in the first few days of parasitism, *T. planipennisi* parasitism induced an overall reduction of the metabolic rate and decrease in feeding activity of parasitized host larvae over their development period. In addition, there was a negative relationship between feeding activity of parasitized hosts and brood sizes of the parasitoid progeny—i.e., the more parasitoid progeny a host larva received, the less feeding activity the host had. These findings suggest that *T. planipennisi* has limited ability to optimize its offspring development and fitness through regulations of the host metabolism and feeding activity and its parasitism reduces feeding damage of parasitized EAB larvae to infested ash trees.

## Introduction

Parasitoid-host interactions form the foundation of biological control strategies against many agriculture and forest insect pests^1,2^. While idiobiont parasitoids, mainly ectoparasitoids, often immediately arrest the development and growth of their hosts upon host attack, koinobiont parasitoids, mainly endoparasitoids, often exert intricate regulations of host physiology, feeding behavior, development, and growth to optimize the host utilization and fitness of the parasitoid offspring. Host regulation by parasitoids involves changes in multiple metabolic pathways of the parasitized hosts, which may be reflected in and measured by host’s respiration rates^3,4^.

The mechanism of host regulation by parasitoids varies with species, and may include the injection of venom, polydnaviruses, or teratocytes when laying eggs on or in their hosts^5–12^. Recent studies indicate that parasitoid life-history attributes, such as offspring brood size, may have profound impacts on host regulation strategies and potentially affect the hosts’ food plant defenses against the herbivorous insect pests^10^. Therefore, understanding the host regulation by the parasitoid is important in developing effective biocontrol strategies against herbivorous insect pests.

*Tetrastichus planipennisi* (Hymenoptera: Eulophidae) is an important natural enemy of the emerald ash borer (EAB), *Agrilus planipennis* (Coleoptera: Buprestidae), in Northeast Asia. Shortly after the EAB invasion of North America, it was introduced to the United States in 2007, along with several other species of hymenopteran parasitoids from EAB’s native range (Northeast Asia) for biological control. It has since become an important biocontrol agent against EAB for protection of ash (*Fraxinus* spp.) trees in North America^13^. *Tetrastichus planipennisi* is a gregarious, koinobiont endoparasitoid, attacking late (3^rd^ to 4^th^) instars of EAB larvae, which feed in the live phloem of ash trunks or branches, making serpentine-like galleries that are filled with larval frass between the cambium and sapwood*.*

Upon laying eggs inside a host larva, *T. planipennisi* does not arrest the host larval growth and development until its larvae reach late instars that consume and fill the interior of the host larval body, giving the cuticle a lumpy, and braided appearance (also termed as the “braided” stage). This process from eggs to “braided” stage, normally takes approximately 9–21 days under normal laboratory rearing conditions^14^. After the “braided” stage, mature instar *T. planipennisi* larvae break free of the host cuticle and pupate inside the host gallery where the parasitoid larvae continue to develop to the adults, which then chew through the bark to exit through small circular exit holes. Each gravid female *T. planipennisi* adult may deposit a clutch of eggs inside a single host larva, whereas a host larva may also receive multiple clutches of eggs from different gravid female wasps^14^. Thus, the brood size of parasitoid offspring inside a parasitized host larva may exhibit high variability, ranging from a few to 10 dozen^14,15^. Currently, no studies have examined if and how *T. planipennisi* parasitism would affect or regulate the metabolism and feeding behavior of the parasitized EAB larvae.

In the present study, we hypothesized that prior to the complete arrest of host development, *T. planipennisi* regulates host metabolism and feeding behavior to optimize its offspring development and fitness. To test this hypothesis, we first compared the respiration rates of parasitized vs unparasitized host larvae at different times (days), and then measured feeding activity of both parasitized and unparasitized host larvae inside their feeding galleries. The brood size of *T. planipennisi* in each parasitized host larva was also recorded and the relationship between parasitoid brood size and feeding activity of parasitized larvae also analyzed.

## Materials and methods

### Parasitoids

All *T. planipennisi* used in the study were reared on EAB larvae infesting green (*Fraxinus pennsylvanica*) or tropical (*F. uhdei*) ash bolts according to methods described in Duan et al.^16^ at the USDA APHIS PPQ Biological Control Production Facility (Brighton, MI) or the USDA, ARS Beneficial Insects Introduction Research Unit (BIIRU) (Newark, DE). Prior to use in experiments, newly emerging (< 1wk old) adult parasitoids (both sexes) were housed in ventilated acrylic cylinders (20 cm height × 12 cm diameter) in walk-in environmental chambers (CTH-1215, Percival Scientific, Perry, IA) at 25 ± 1.5 °C, 65 ± 10% RH, and 16: 8 h (L: D) photoperiod.

### Host larvae

All EAB larvae used in the study were early 4th instars reared with greenhouse-grown tropical ash bolts (~ 2 cm diam and 20 cm length) at BIIRU according to methods described in Duan et al.^17^. Prior to use in experiments, all EAB larvae were removed from the rearing ash bolts, early 4th instars were then selected based on the width (3–4 mm) of feeding galleries at their head positions and immediately inserted into newly cut tropical ash bolts (same size as the rearing bolts, but with one larva per bolt) according to methods described in Duan and Oppel^15^.

### Host exposure to parasitoids

To produce parasitized EAB larvae for experiments, ash bolts containing inserted-EAB larvae (one larva per bolt) were exposed under the normal rearing condition to naïve gravid females of *T. planipennisi* adults at 10: 1 parasitoid to host ratio in a rearing jar (3.5 Liter) for 48 h to ensure high rates of parasitism^18^. For the respirometry test, a total of 32 ash bolts each containing a single inserted EAB larva were exposed to the parasitoids at various times; 11 ash bolts containing inserted EAB larvae (one larva per bolt) not exposed to the parasitoids were used as negative control (i.e., healthy, unparasitized host larvae). For measurement of feeding activity, a total of 49 ash bolts were exposed to the parasitoids and 28 ash bolts not exposed to parasitoid were used for negative control. All test EAB larvae were dissected out of the ash bolts at various times and confirmed for parasitism by *T. planipennisi* for both respirometry tests and feeding activity measurements. Unhealthy and dead or dying EAB larvae (from ~ 20% of ash bolts) were not used for both respirometry test and feeding activity measurements.

### Respirometry test

Parasitized host larvae were dissected from exposed ash bolts every 2 days after the 48-h parasitoid exposure treatment, confirmed for parasitism, and immediately tested for carbon dioxide (CO_2_) emission, water (H_2_O) loss, and activity (or movement) with a flow-through CO_2_/H_2_O analyzer equipped with an infrared red-light activity sensor (Li-7000, Li-COR, Inc., Lincoln, NE). At each test time, EAB larvae of the same stage were dissected out from the control ash bolts (not exposed to parasitoids) and tested with the same CO_2_/H_2_O analyzer and Infrared light activity sensor. Due to shortage of suitable stages of EAB larvae, we missed respirometry tests with unparasitized (healthy) EAB larvae at days 6 and 8 after the parasitoid exposure treatments but resumed the regular tests with both unparasitized and parasitized EAB larvae thereafter.

The tests were conducted at room temperature according to methods described in Zheng et al.^19,20^. The flow rate used for all tests was 4.2 ml min^−1^, which was controlled by a Cole-Parmer flow meter (Model 32003-04) and measured by a GC flow meter (VKIT GFM 3). During each test, a blank chamber (baseline measurement) was run for 0.5 h before testing parasitized or unparasitized samples, respectively. The CO_2_ emission and water loss of each test larva inside the test chamber were recorded at 5 s intervals for 1.5 h. All test larvae were weighed before and after the test to obtain the total amount of body mass loss during 1.5 h recording. A total of 17 parasitized EAB larvae and 10 unparasitized host larvae of the same stage or age were tested at various times with a flow-through CO_2_/H_2_O analyzer. The activity or movement of each test insect were also recorded by the Infrared light activity sensor at the same rate as CO_2_ emission and water loss.

ExpeData 1.0.24 (Sable Systems, Inc., Las Vegas, NV) was used to calculate and analyze the CO_2_ emission and water loss rates in both parasitized and unparasitized EAB larvae according to methods described in Zheng et al.^20^. Baseline drift was corrected according to the measurements at the beginning of each test^21^. Before data analysis using ExpeData, the first 0.5 h of the sample recording was deleted because of interference from placement of test larva inside test chamber (i.e., from opening and closing of the test chamber). The CO_2_ emission in µl h^−1^ was calculated by multiplying instantaneous CO_2_ emissions and the air flow rate^22^ according to procedures described in the software (ExpeData 1.0.24). The standard metabolic rate (VCO_2_) was estimated as the mean CO_2_ emission rate (µl h^−1^) over 1 h of gas exchange. Water loss data were converted to mg h^−1^^20,23^. Average activity or movement signals (unitless) was calculated for each test insect after excluding the first 0.5 h of activity recording in ExpeData 1.0.24.

### Measurement of feeding activity

**Figure 1:**
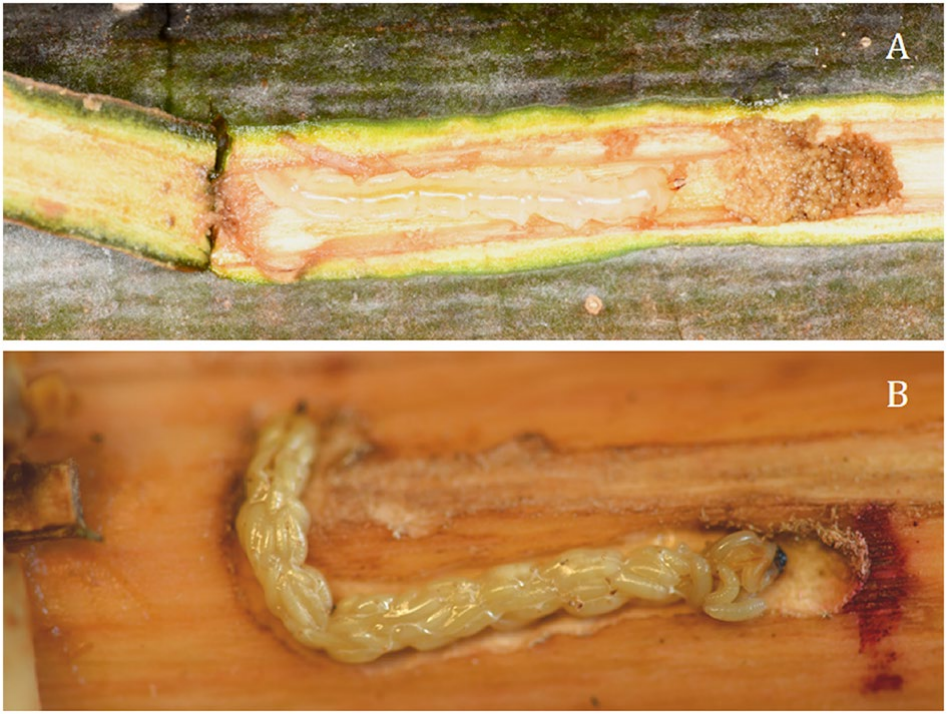
Emerald ash borer (EAB) parasitized by *Tetrastichus planipennisi*: (**A**) Inserted EAB larva and its gallery, and (**B**) Braided stage of *T. planipennisi* larvae.

Beginning from 2 d post-parasitism (or parasitoid exposure), each ash bolt was peeled by gently pulling the insertion bark flap and nearby bark tissues (if needed) every 2 days to reveal the EAB larva and its gallery (Fig. 1A). Once the larva and its gallery were exposed, we measured both length and width of the larval gallery using a string at the insertion (feeding-initiation) point and the top of the gallery where the head of the larva was located, the length of string was then measured using digital caliper (UltraTech, General Tools, USA). The surface area of the larval gallery was then calculated as a trapezoid for each larva at various times (every 2 days after parasitism), i.e., the surface area measured each time represented the completely new gallery area formed during the 2-day feeding period of EAB larvae and excluded any surface areas of galleries that had been previously measured. In addition, the frass of each exposed larva was collected onto a square piece of aluminum foil (5 cm × 5 cm) using soft forceps and brush. The frass samples were then dried inside an oven at 60 °C for 2–3 days and then weighed using an analytical balance (± 0.01 mg, AB135-S/FACT; Mettler Toledo, Switzerland).

Measurement of EAB larval feeding activity stopped 14 days post parasitism, at which time a majority parasitized EAB larvae had stopped feeding because the parasitoid larvae completely consumed the soft tissues of the host larva and advanced to braided stage (Fig. 1B). Each of those parasitized host larvae was then moved individually to a Falcon Petri dish (diameter = 4 cm) containing a moist filter paper and incubated under normal rearing conditions until parasitoid adults emerged. The number of *T. planipennisi* offspring (brood size) produced from each host larva was also recorded when the parasitoid larvae advanced to free living stages. A total of 25 parasitized larvae and 36 unparasitized larvae were measured for their feeding activities (gallery surface area and frass weight) in this experiment.

### Statistical analysis

All data were assessed for homogeneity with Bartlett tests and normality with Shapiro–Wilk tests prior to linear regression analyses. The goodness of fit for each linear regression model was assessed based on the residual plots. Significant heterogeneities were observed in our data between parasitized and unparasitized host larvae as well as among different observation times (different days post parasitism). Consequently, we needed to include the interaction between parasitism status and observation time (treated as a continuous variable) in our general linear model (GLM) for ANOVA: Y_i_ = Parasitism status + Observsation time + Parasitism status × Observation time + Error, where Yi is the responsive variable (e.g., metabolic rate, water loss, body mass loss, etc.).

To further illustrate the interactions between parasitism status and observation time, we used simple linear regression to detect significance in relationships or changes of test larval metabolic rate, activity signals, water loss (during 1 h recording), and body mass loss (during 1.5 h recording) in relation to days after parasitoid exposure treatments for both parasitized and unparasitized host larvae. Similar linear regression procedures were also used to detect differences between parasitized and unparasitized host larvae in feeding activities – gallery surface area and frass weight in relation to days after parasitoid exposure treatments.

In addition, we used mixed linear model by including the observation time as repeated measure variable to detect any effect of parasitoid progeny brood size on feeding activities (gallery surface area and frass weight) of parasitized host larvae. All the analyses were conducted with the Fit Model platform in JMP Pro 17 (SAS 2023).

## Results

### Respiration rates of parasitized and unparasitized larvae

**Figure 2:**
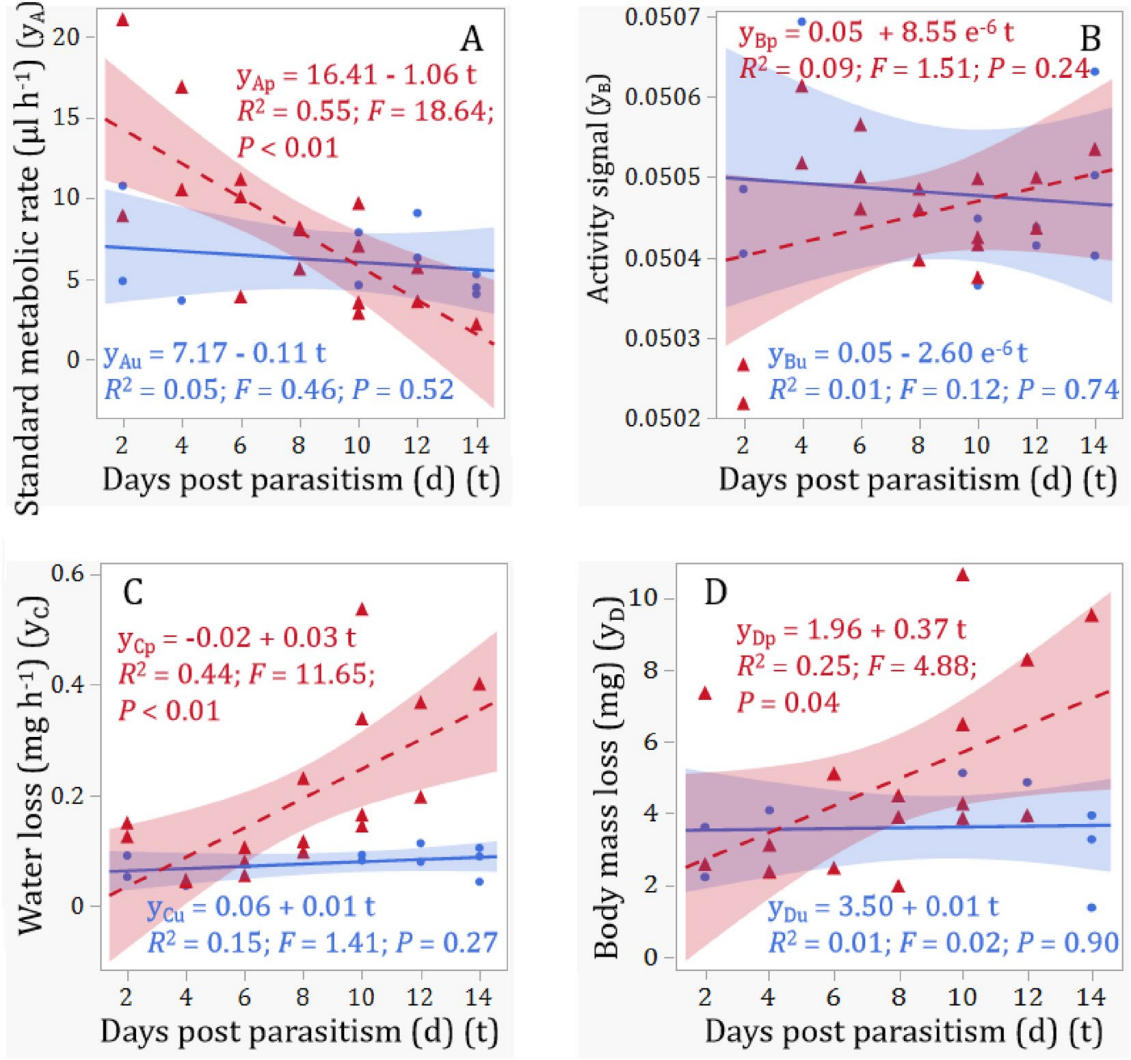
Respirometry tests with parasitized and unparasitized EAB larvae over different days post parasitism: (**A**) standard metabolic rate during 1 h recording, (**B**) activity signal during 1 h recording, (**C**) water loss during 1 h recording, and (**D**) body mass loss during 1.5 h recording. Red dashed lines, triangles, and 95% confidence interval (shaded red area) are for parasitized larvae and blue solid lines, dots, and 95% confidence interval (shaded blue area) are for unparasitized controls.

There was no significant main effect of parasitism status on the standard metabolic rate [i.e., mean CO_2_ emission rate (µl h^−1^)], but there were significant main effects of observation time as well as significant interactions between parasitism status and observation time (Table 1). Further simple linear regression analyses indicated that there were no significant changes in the standard metabolic rate in unparasitized EAB larvae over the observation period (2–14 days post parasitism) (*P* = 0.52; Fig. 2A), whereas the standard metabolic rate decreased significantly from 21.0946 µl h^−1^ at day 2 post parasitism to 2.1690 µl h^−1^ at day 14 in parasitized EAB larvae (*P* < 0.01; Fig. 2A). In addition, the metabolic rate appeared to be higher in parasitized EAB larvae than in unparasitized larvae from day 2 to day 6 post parasitism, but lower from days 12 to 14 post parasitism (Fig. 2A).

**Table 1:** Effects of parasitism status, observation time, and their interactions on each responsive variable from GLM (multiple linear regression) analyses.

Experiment	Responsive variables	Predictor variables	*df*	*F* value	*P* value
Respirometry tests	Standard metabolic rate	Parasitism status	1, 26	1.0510	0.3159
		Observation time	1, 26	14.3712	0.0009*
		Parasitism status × Observation time	1, 26	9.3570	0.0056*
	Activity signal	Parasitism status	1, 26	0.3579	0.5555
		Observation time	1, 26	0.3448	0.5628
		Parasitism status × Observation time	1, 26	1.2139	0.2820
	Water loss	Parasitism status	1, 26	12.1759	0.0020*
		Observation time	1, 26	10.4948	0.0036*
		Parasitism status × Observation time	1, 26	7.7130	0.0107*
	Body mass loss	Parasitism status	1, 26	3.2034	0.0867
		Observation time	1, 26	3.6352	0.0691
		Parasitism status × Observation time	1, 26	3.2479	0.0846
Feeding activity measurement	Gallery surface area	Parasitism status	1, 327	6.0395	0.0145*
		Observation time	1, 327	12.8460	0.0004*
		Parasitism status × Observation time	1, 327	5.0296	0.0256*
	Frass weight	Parasitism status	1, 327	0.8361	0.3612
		Observation time	1, 327	10.6923	0.0012*
		Parasitism status × Observation time	1, 327	9.9702	0.0017*

Asterisk represents significance at the level of 5%.

ANOVA detected no significant main effects of parasitism status, observation time as well as their interactions on activity signals of tested EAB larvae (Table 1). Further simple linear analyses revealed no significant changes in activity signals in both parasitized and unparasitized host larvae over the testing period (*P*s > 0.23; Fig. 2B).

While there were no significant main effects of parasitism status, observation time, and their interactions on the body mass loss of tested EAB larvae (*P*s > 0.06, Table 1), significant main effects of parasitism status, observation time, and their interactions on water loss of tested EAB larvae were detected (*P*s < 0.02. Table 1). Simple linear regression analyses further revealed that both the water loss (Fig. 2C) and body mass loss (Fig. 2D) of parasitized EAB larvae increased significantly from days 2 to 14 post parasitism (*P*s < 0.05), whereas there were no significant changes in both water loss and body mass loss in the control treatment (healthy, unparasitized EAB larvae) over the testing period (*P*s > 0.25; Fig. 2C and D).

### Feeding activities of parasitized and unparasitized larvae

**Figure 3:**
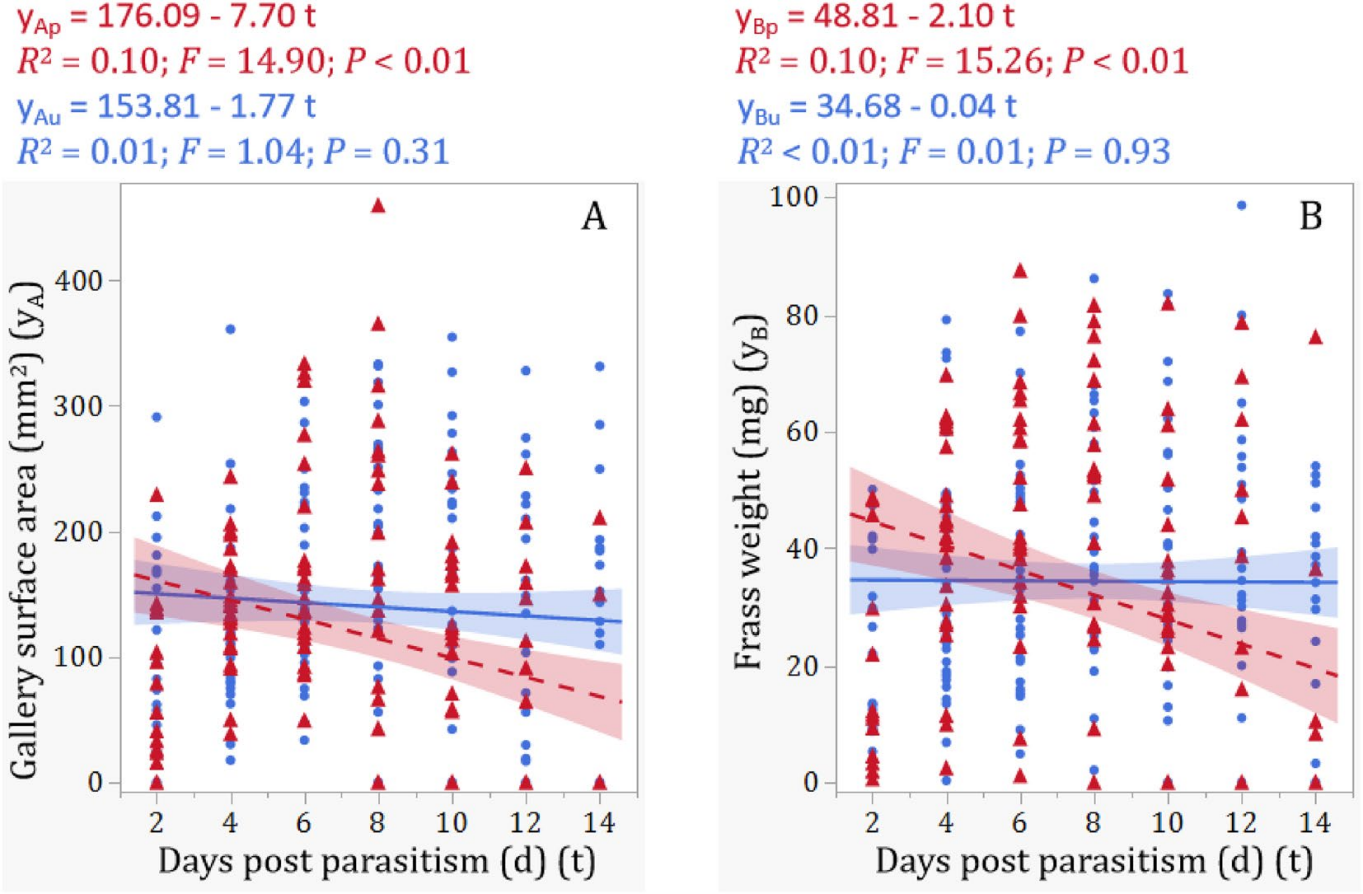
Gallery surface area (**A**) and frass weight (**B**) of parasitized and unparasitized EAB larvae over different days post parasitism. The range (colored) of each regression line represents the 95% confidence interval. Red dashed lines, triangles, and range are for parasitized larvae and blue solid lines, dots, and range are for unparasitized controls.

**Figure 4:**
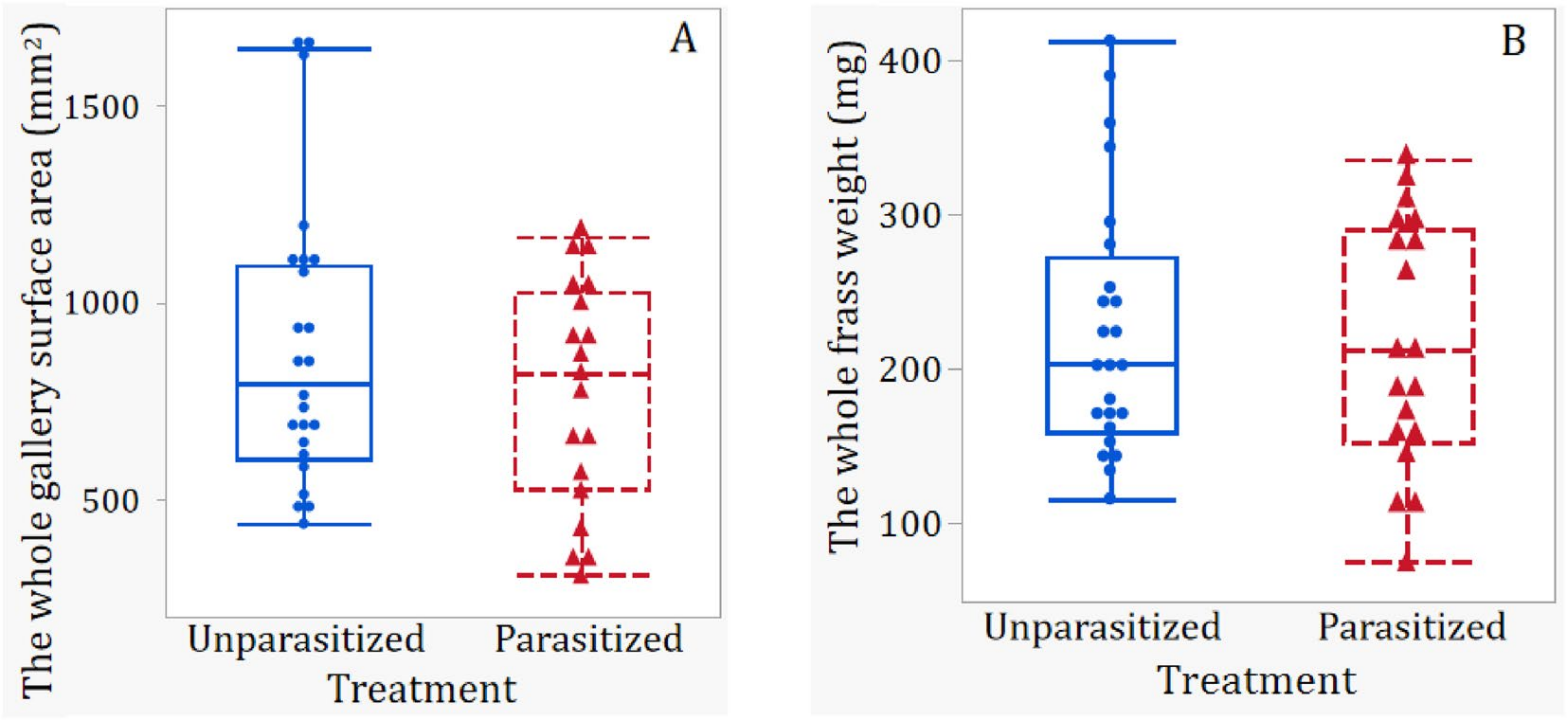
Total gallery surface area (**A**) and frass weight (**B**) of each parasitized and unparasitized EAB larvae for the entire testing period (days 2 to 14 post parasitism). Red dashed lines and triangles are for parasitized larvae and blue solid lines and dots are for unparasitized controls.

There were significant main effects of parasitism status, observation time, and their interactions on the surface area (mm^2^) of galleries made by observed EAB larvae (*P*s < 0.03, Table 1). While parasitism status did not significantly affect the frass weight from observed EAB larvae (*P* = 0.3612), there were significant effects of observation time as well as the interaction between parasitism status and observation time (*P* < 0.01, Table 1). Simple linear analyses further revealed that both surface area of larval galleries and dry frass weight decreased significantly with observation time for parasitized larvae (*P*s < 0.01; Fig. 3A and B), but did not change significantly with observation time for the unparasitized (control) EAB larvae (*P* > 0.3, Fig. 3A and B). Further mean comparisons indicated that the total surface area of feeding galleries made by parasitized EAB larvae was significantly less than that of unparasitized larvae (*t* = 2.46; *P* = 0.0145, Fig. 4A), whereas the mean dry frass weight excreted per larva was not significantly lower for parasitized larvae than the control larvae (*t* = 0.91; *P* = 0.3612; Fig. 4B), possibly due to large variations in dry frass weight among test insects.

**Figure 5:**
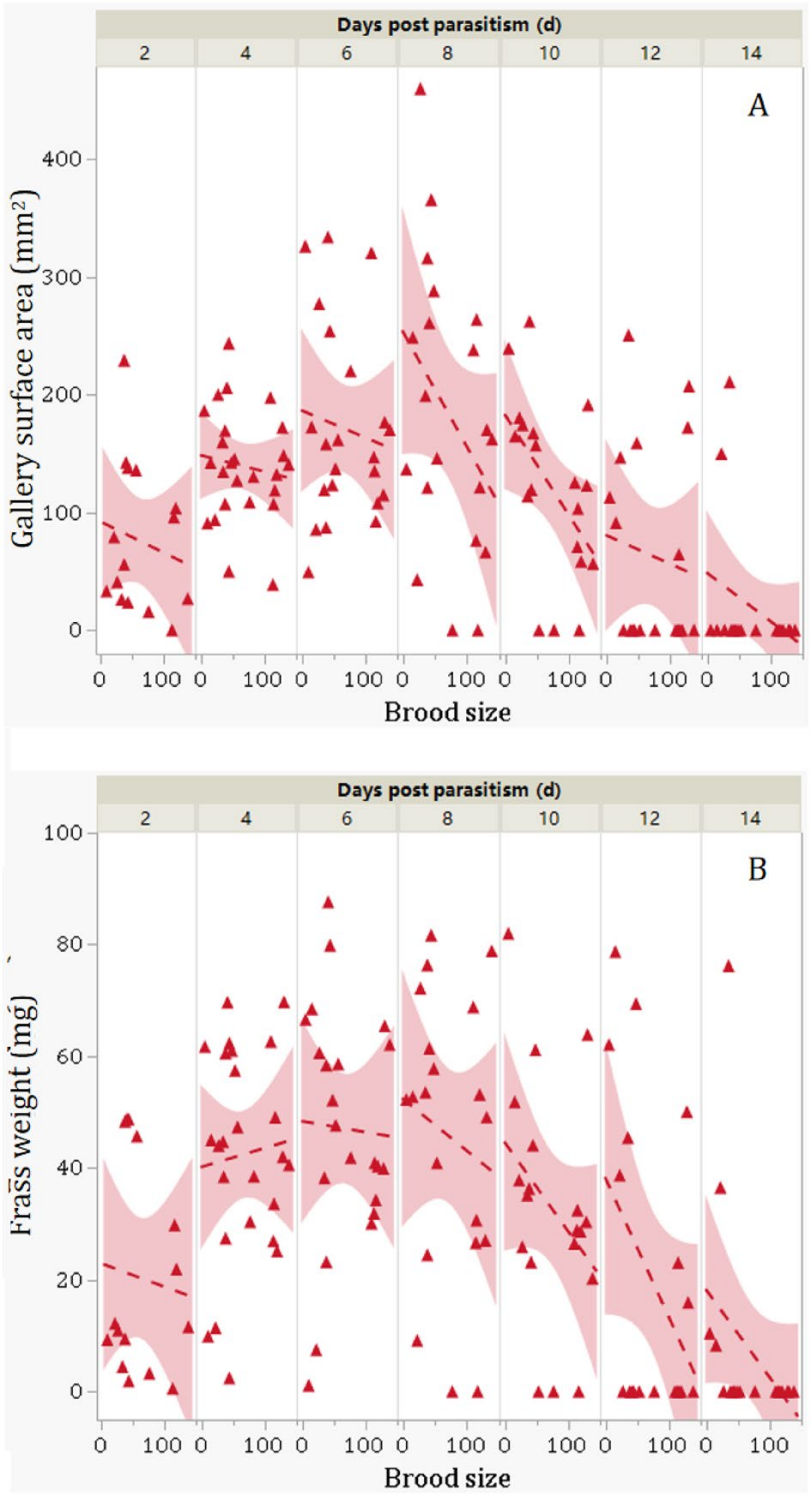
Relationship of parasitoid progeny brood size and gallery surface area (**A**) and frass weight (**B**) of parasitized EAB larvae based on mixed linear model by including the observation time as the repeated measure variable.

For the parasitized EAB larvae, we noticed large variations in the brood size of parasitoid progeny, which ranged from 5 to 139 per parasitized host larva. Further mixed linear model analyses showed significantly negative relationship between the parasitoid progeny brood size and the surface area of host larval gallery (*df* = 1, 138; *F* = 6.5021; *P* = 0.0119) (Fig. 5A) or dry frass weight (*df* = 1, 138; *F* = 4.2589; *P* = 0.0409) (Fig. 5B). These results indicated that parasitoid progeny brood sizes negatively affected feeding activities of parasitized host larvae.

## Discussion

Parasitoids can elicit diverse changes in physiological and/or behavioral responses of the host to ensure parasitism success and establish a host environment tailored to the requirements of their offspring^24–26^. In the present study, although parasitized host larvae showed increased metabolic rate in the first few days of post parasitism, *T. planipennisi* parasitism induced an overall reduction of the metabolic rate and feeding activity of parasitized host larvae over a period from days 2 to 14 post parasitism. In addition, there was a negative relationship between feeding activity of the parasitized host and the brood size of the parasitoid progeny inside the host—i.e., the more parasitoid progeny a host larva received, the less feeding activity the larva had. These findings indicate that *T. planipennisi* may have limited ability to optimize its offspring development and fitness through regulations of the host metabolism and feeding activity via larval parasitism.

**Figure 6:**
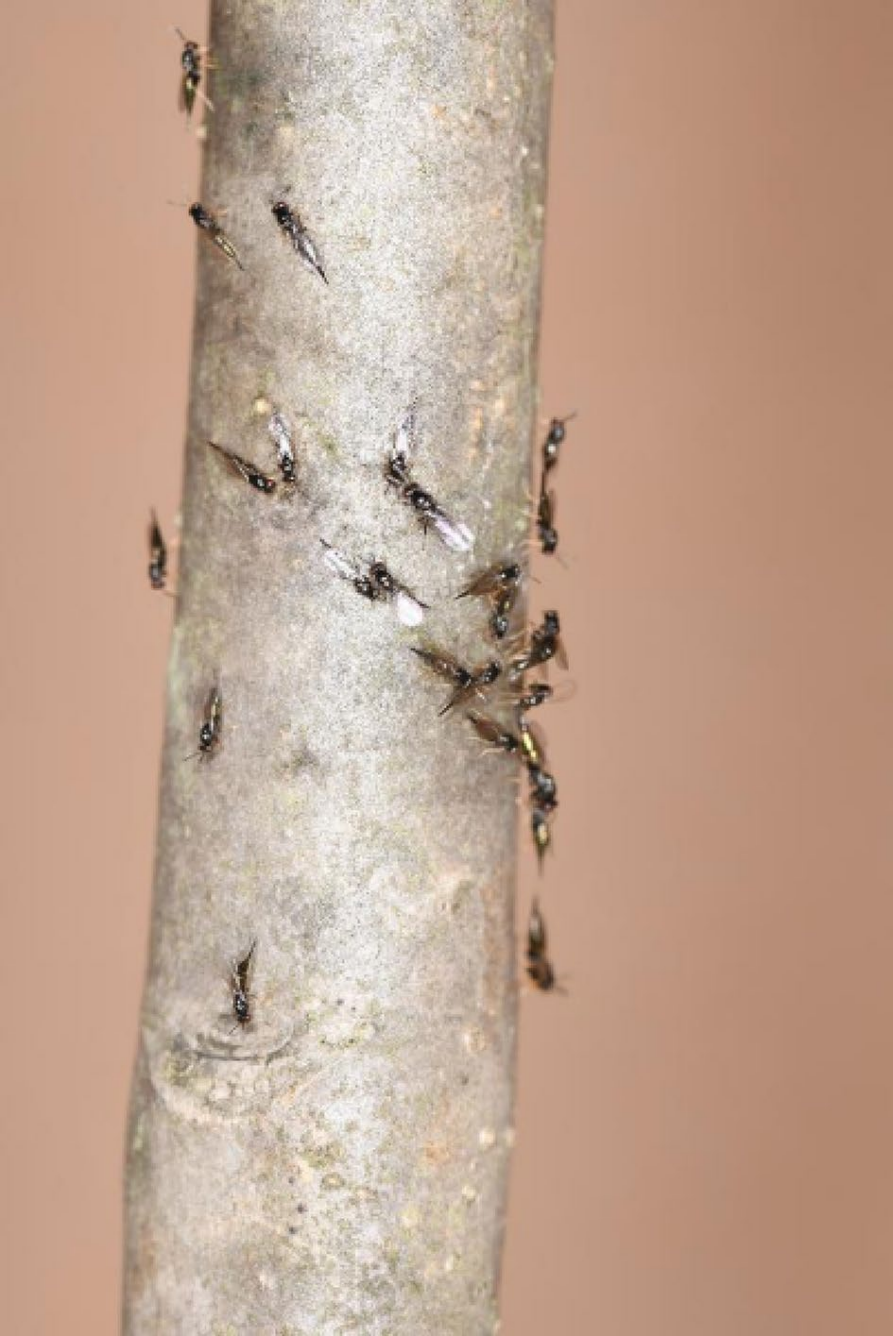
Multiple female *T. planipennisi* probing a small ash log containing a single EAB larva.

It is well documented that parasitoids must rely on resources obtained from single parasitized hosts for successful completion of larval development^27^ because of their inability to obtain essential nutrients independently after parasitism. Limited by the host resource, parental female parasitoids must determine the brood size and host use strategy when attacking a potential host to balance the fertility and food resource^28^. Therefore, like many other species of gregarious parasitoids^5,10^, optimization of offspring development and fitness by *T. planipennisi* must be accomplished at the time of parasitism primarily by parental adult wasps through selection of suitable stages of host larvae (late 3rd or 4th instars) for parasitism and allocation of adequate number of eggs and progeny sexes to each parasitized host larva^14,15^. It is important to note that the large variations in brood sizes of *T. planipennisi* progeny in the present study could be attributed to the influence from factors such as parasitoid and host densities, exposure time, and/or eggloads of parental female wasps. This has been shown in several laboratory studies^14,15,29^ as well as our own observation that multiple female *T. planipennisi* adults probe a small ash log containing a single EAB larva (Fig. 6).

Among the numerous metabolic processes, gaseous metabolism, specifically referring to the metabolic rate, respiration, and respiratory water loss, is the prominent and fundamental pathway for parasitoid host regulation upon parasitism. It is well documented that parasitism by some parasitoids like *Cotesia congregata* (Hymenoptera: Braconidae)^24^, *Ampulex compressa* (Hymenoptera: Ampulicidae)^30^, and *Cotesia vestalis* (Hymenoptera: Braconidae)^31^ may lead immediate decreases in the respiration and metabolic rates of parasitized hosts, whereas parasitism by other parasitoids such as *Misotermes mindeni* (Diptera: Phoridae) can increase the metabolic rate of parasitized host larvae^32^. The possible reasons for the differences in host metabolic responses to parasitism may be related to different host-utilization strategies by different parasitoid species^10,32^.

To prevent excessive desiccation and minimize stress responses triggered by dehydration^32,33^, parasitoids may maintain the host’s water balance before they become independent of their host insects. In our study, the respiratory water loss of parasitized EAB larvae at first several days post parasitism was consistent with that of unparasitized larvae. The significantly higher metabolic rate observed in parasitized host larvae (in comparison to the unparasitized control host larvae) during the first few days may reflect a metabolic cost of the physiological reaction to parasitism by the host larvae. However, parasitized host larvae did not show a reduced water loss rate or body weight loss during this early period of parasitism is intriguing. These results seem contradictory to the common expectations that higher respiration and metabolic rates would result in increased water loss^34,35^. These unexpected observations may be attributed to the water-conserving function of the integument of EAB larvae before parasitoids reach maturity and break free of the host, like in other insects^32,36^. Nevertheless, further studies are needed to address unexpected results in water and host larval weight loss during the respirometry test.

Together, our findings may have several implications for EAB biocontrol with *T. planipennisi*. First, parasitism by *T. planipennisi* is not likely to increase feeding damage of parasitized EAB larvae to host phloem issues. Rather, it reduces the overall feeding activity of parasitized larvae and thus limits the damage to host’s food plant tissues. Second, optimization of its offspring development and fitness by *T. planipennis* relies on selection of suitable stages of EAB larvae, not by regulations of the host metabolism and feeding activity via larval parasitism. Thus, the availability and abundance of suitable stages of host EAB larvae (3rd to 4th instars) are critical for successful parasitoid progeny production and population growth in the field as well as in laboratory rearing. Thirdly, as there was a negative relationship between feeding activity of the parasitized host and the brood size of the parasitoid progeny inside the host, superparasitism may result in reduction of the damage caused by the parasitized larvae to their host plants and thus benefit EAB biocontrol.

## Data Availability

Data will be available upon the publication of the manuscript through the Ag Data Commons (National Agricultural Library, USDA Agricultural Research Service).
